# Similar prevalence of hepatic steatosis among patients with chronic hepatitis C with and without HIV coinfection

**DOI:** 10.1038/s41598-020-62671-y

**Published:** 2020-04-21

**Authors:** M. Fernandez-Fuertes, J. Macías, A. Corma-Gómez, P. Rincón, N. Merchante, J. Gómez-Mateos, J. A. Pineda, L. M. Real

**Affiliations:** Unit of Infectious Diseases and Microbiology, Hospital Universitario Virgen de Valme, Sevilla, 41014 Spain

**Keywords:** Viral hepatitis, HIV infections

## Abstract

Hepatic steatosis (HS) is frequently observed in HIV-infected patients. It is not known whether HIV infection is an independent risk factor for HS development. We aimed to analyze whether HIV coinfection was associated with a higher frequency of HS in patients with chronic hepatitis C. This was a retrospective cross-sectional study. 574 subjects with chronic hepatitis C virus (HCV) infection were included, 246 (43%) of them coinfected with HIV. All of them underwent transient elastography with controlled attenuation parameter (CAP) measurement. HS was defined as CAP ≥ 248 dB/m. 147 individuals (45%) showed HS in the HCV-monoinfected group and 100 (40.7%) in the HIV/HCV-coinfected group (p = 0.318). HS was associated with body mass index (BMI) [<25 Kg/m^2^ vs. ≥25 Kg/m^2^, 67 (23.5%) vs. 171 (62.9%); p = 0.001], with plasma HDL-cholesterol [<50 mg/dL vs. ≥50 mg/dL, 122 (48.6%) vs. 95 (37.5%), p = 0.012], with plasma triglycerides [<150 mg/dL vs. ≥150 mg/dL, 168 (40.2%) vs. 65 (52.4%); p = 0.016] and with plasma total cholesterol [<200 mg/dL vs. ≥200 mg/dL, 181 (41%) vs. 53 (52.5%); p = 0.035]. In the multivariate analysis, HS was associated with BMI [adjusted OR (AOR) = 1.264 (1.194–1.339); p = 0.001], age [AOR = 1.029 (1.001–1.058); p = 0.047] and HCV genotype 3 infection [AOR = 1.901 (1.081–2.594); p = 0.026]. HIV coinfection was not associated with HS [AOR = 1.166 (0.719–1.892); p = 0.534]. In conclusion, HIV coinfection is not related with an increased frequency of HS in HCV-infected patients.

## Introduction

The main causes of hepatic steatosis (HS) are metabolic factors^1^, alcohol use^2^ and HCV infection, particularly HCV genotype 3^3,4^. Non-alcoholic fatty liver disease (NAFLD), HS in the absence of other causes than metabolic factors, is recognized as one of the most common liver diseases worldwide with an estimated prevalence of 25% among the general population in Western countries^5,6^. NAFLD has a spectrum of liver disease ranging from simple fatty liver to nonalcoholic steatohepatitis (NASH), fibrosis and cirrhosis^7–10^.

In HIV-uninfected patients, the prevalence and risk factors of NAFLD and its complications have been evaluated, specifically in industrialized countries^6,8,11–13^. In HIV infection, studies carried out in unselected patients with or without HCV coinfection have reported a prevalence of HS, determined by the controlled attenuation parameter (CAP), of approximately 40%^14–17^. This rate nearly doubles that found of the general population in similar areas^5^ and it is closer to the NAFLD prevalence observed in obesity and type 2 diabetes mellitus^8^. Despite this difference between HIV-infected patients and the general population, there are very few direct comparisons of HS frequency between individuals with and without HIV infection. Indeed, a study comparing men who have sex with men (MSM) with and without HIV infection found a lower prevalence of HS in HIV-infected MSM than in MSM without HIV infection^18^. On the contrary, two case-control studies conducted in small samples found higher frequencies of HS in HIV-infected individuals than in controls^19,20^. Because of this, the actual impact of HIV infection on the risk of HS still remains unknown.

Among HIV-infected patients with chronic hepatitis C, the frequency of HS estimated by liver biopsy showed a wide range from 23% to 72%^21^. In a metanalysis of HCV-infected patients who underwent liver biopsy, the rates of HS were similar in HIV/HCV-coinfected and HCV-monoinfected subjects^21^. However, patients undergoing a liver biopsy are not representative of the overall population with HCV infection. Thus, information provided by this study on the risk of HS inherent to HIV infection is very limited. Because of this, comparative studies of the HS prevalence conducted in unselected populations of HCV-infected patients with and without HIV coinfection are required. Non-invasive techniques may allow these studies, as the overall HCV-infected population, irrespective the estimated liver stage or the therapy perspectives, may be included^9,10^. This kind of studies may provide very valuable data on the risk of HS due to HIV infection minimizing the possible confounding effect of the HCV coinfection.

Because of these, we aimed to compare the prevalence of HS, evaluated using CAP, in patients with chronic HCV infection, with and without HIV coinfection, in order to appraise the effect of HIV infection on the HS presence in this setting.

## Results

### Characteristics of the study population

Five hundred and ninety-eight consecutive patients fulfilled the inclusion criteria. A reliable elastography result could not be obtained in 24 (4%) of them. Thus, 574 patients were finally analyzed. Among them, 328 (57.1%) were HCV-monoinfected patients and 246 (42.9%) were HIV/HCV-coinfected individuals. The demographic, anthropometric and laboratory characteristics of these patients are shown in Table 1. BMI and plasma total cholesterol, HDL cholesterol and LDL cholesterol values were lower in HIV/HCV-coinfected than in HCV-monoinfected patients (Table 1). Nearly all of HIV/HCV-coinfected patients were under antiretroviral therapy [241 (99.2%)] with undetectable viral load in 78.9% of the cases (Table 1).

**Table 1:** Characteristics of study populations (N = 574).

Characteristics	HCV monoinfection (N = 328)	HIV/HCV coinfection (N = 246)	p-value
Male sex, n (%)	271 (82.6)	214 (87)	0.152
Age (years)*	52 (48–57)	53 (49–56)	0.695
IDU^a^, n (%)	205 (66.3)	209 (87.1)	0.001
Alcohol intake ≥50 g/d, n (%)	168 (51.2)	60 (24.4)	<0.001
HCV Genotype 3 infection, n (%)	54 (16.5)	43 (17.5)	0.748
BMI^b^ (kg/m^2^)*	25.9 (22.6–28.5)	24 (21.3–27)	0.001
BMI^b^ (kg/m^2^), n (%)			
18–25	141 (44.3)	144 (60.3)	0.004
26–30	123 (38.7)	67 (28)	
31–35	41 (12.9)	18 (7.5)	
>35	11 (3.5)	9 (3.8)	
Fasting plasma glucose^c^ ≥100 mg/dL, n (%)	72 (24.4)	75 (30.5)	0.113
Plasma triglycerides^d^ ≥150 mg/dL, n (%)	42 (14.1)	82 (33.5)	0.001
Plasma total cholesterol^e^ (mg/dL)*	165 (145–199)	156 (137–182)	0.001
Plasma HDL-cholesterol^f^ (mg/dL)*	52.5 (41.5–65.4)	47.5 (37.6–59.0)	0.003
Plasma LDL-cholesterol^g^ (mg/dL)*	91 (71–115)	76 (61–99)	0.001
LS (kPa)*	7.3 (5.4–13.3)	9.7 (6.9–16.9)	0.001
Cirrhosis, n (%)	78 (23.8)	80 (32.5)	0.020
HbsAg^h^+, n (%)	1 (0.3)	8 (3.3)	0.001
CD4 cell counts/mm^3^)^I^ *	—	486 (310.5–726.0)	
Plasma HIV-RNA < 50 copies/mL, n (%)	—	194 (78.9)	
ART^j^, n (%)	—	241 (99.2)	

*Median (Q1-Q3).

^a^Available data for 549 patient; ^b^Available data for 557 patients; ^c^Available data for 541 patients; ^d^Available data for 542 patients; ^e^Available data for 543 patients; ^f^Available data for 504 patients; ^g^Available data for 505 patients; ^h^Available data for 562 patients; ^i^Available data for 245 patients; ^j^Available data for 243 patients.

Abbreviations: HCV: hepatitis C virus; HIV: human immunodeficiency virus; IDU: injecting drug users; BMI: Body mass index; HDL: High-density lipoprotein; LDL: Low-density lipoprotein; LS: Liver Stiffness; ART: antiretroviral therapy.

### Prevalence of HS according to HIV infection status

**Figure 1:**
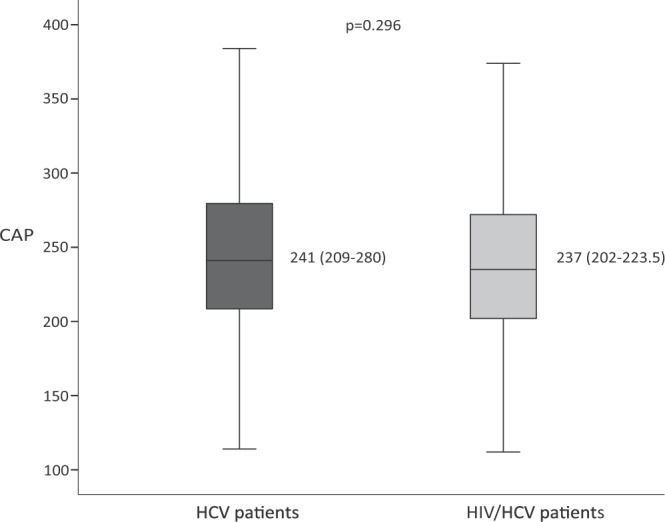
CAP median and interquartile range in both HCV-infected and HIV/HCV-coinfected group of patients.

**Figure 2:**
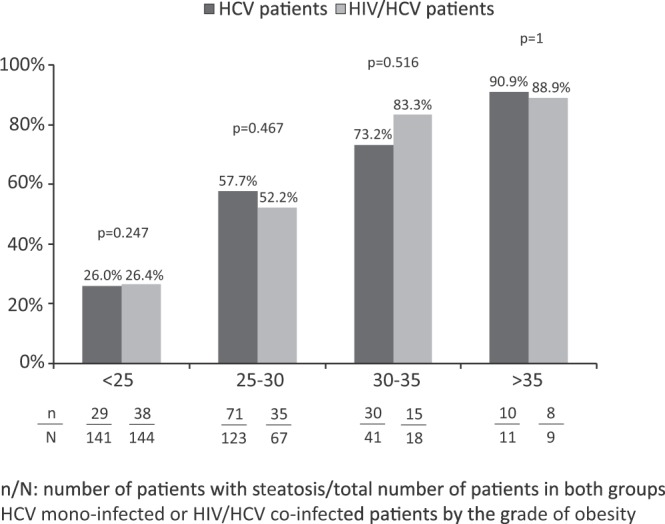
Frequency of steatosis in HCV-infected and HIV/HCV-coinfected populations classified according to the grade of obesity.

Median CAP was 241 (209–280) dB/m among HCV-infected patients and 237 (202–273) dB/m among HIV/HCV-coinfected patients, (p = 0.296) (Fig. 1). The frequency of HS (CAP ≥ 248 dB/m) in HCV-monoinfected patients was 147 (44.8%) and in HIV/HCV-coinfected patients was 105 (40.7%) (p = 0.318). Eighty-three (25.3%) individuals without HIV infection showed severe steatosis compared to 51 (20.7%) among those with HIV coinfection (p = 0.2). There were no differences in the prevalence of steatosis between the group with and without HIV coinfection by BMI category (Fig. 2). In an analysis excluding patients with alcohol intake ≥50 g/d, there were no differences between the two study groups in the median CAP values nor in the prevalence of HS (Supplementary Fig. S1).

Matching HIV/HCV-coinfected patients with HCV-monoinfected individuals by age, sex and BMI (Supplementary Table 1), the prevalence of HS for the HIV/HCV-coinfected group was 100 (40.7%) and 96 (39.0%) for the HCV-monoinfected group (p = 1.000).

### Factors associated with the presence of HS

In the univariate analysis, history of injecting drugs, alcohol intake, plasma triglycerides, BMI, plasma total cholesterol and plasma HDL cholesterol were related with HS, whereas HIV coinfection was not (Table 2). In the multivariate logistic analysis, BMI, age and genotype 3 infection were independently associated with HS. HIV coinfection was not related with HS (Table 2).

**Table 2:** Univariate and multivariate analysis of factors associated with steatosis.

Parameter	No.	Frequency of steatosis** N (%)	p univariate	AOR (95% CI)	p multivariate
Sex					
Female	89	37 (41.6%)	0.762	1.129 (0.597–2.134)	0.709
Male	485	210 (43.3%)			
Age*					
<52	238	92 (38.7%)	0.075	1.030 (1.001–1.060)	**0.040**
≥52	336	155 (46.1%)			
Way of HCV infection					
IDU^a^	414	167 (40.3%)	0.058	0.923 (0.541–1.572)	0.767
Other	135	67 (49.6%)			
HIV					
Positive	246	100 (40.7%)	0.318	1.181 (0.727–1.918)	0.502
Negative	328	147 (44.8%)			
HCV genotype					
3	97	49 (50.5%)	0.102	1.952 (1.106–3.445)	0.021
Other	477	198 (41.5%)			
Alcohol intake (g/day)					
<50	346	134 (38.7%)	0.01	1.395 (0.882–2.208)	0.155
≥50	228	113 (49.6%)			
FPG (mg/dL)*^c^					
<100	394	163 (41.4%)	0.148	1.003 (0.993–1.013)	0.537
≥100	147	71 (48.3%)			
Plasma triglycerides (mg/dL)*^d^					
<150	418	168 (40.2%)	0.016	1.003 (0.999–1.007)	0.130
≥150	124	65 (52.4%)			
BMI (kg/m^2^)*^b^					
<25	285	67 (23.5%)	<0.001	1.266 (1.195–1.340)	**<0.001**
≥25	272	171 (62.9%)			
Plasma total cholesterol (mg/dL)*^e^					
<200	442	181 (41%)	0.035	1.002 (0.995–1.008)	0.562
≥200	101	53 (52.5%)			
Plasma HDL cholesterol (mg/dL)*^f^					
<50	251	122 (48.6%)	0.012	0.997 (0.986–1.008)	0.623
≥50	253	95 (37.5%)			
Plasma LDL cholesterol (mg/dL)^g^					
<160	487	211 (43.3%)	0.709	—	—
≥160	18	7 (38.9%)			
Cirrhosis*					
Yes	416	177 (42.5%)	0.017	0.810 (0.495–1.323)	0.400
No	158	70 (44.3%)			

*Introduced as a continuous variable in the multivariate analysis.

**CAP ≥ 248 dB/m.

^a^Available data for 549 patients; ^b^Available data for 557 patients; ^c^Available data for 541 patients; ^d^Available data for 542 patients; ^e^Available data for 543 patients; ^f^Available data for 544 patients; ^g^Available data for 505 patients.

Abbreviations: AOR: Adjusted Odds Ratio; HCV: hepatitis C virus; HIV: human immunodeficiency virus; IDU: injecting drug users; FPG: Fasting plasma glucose; BMI: Body mass index; HDL: High-density lipoprotein; LDL: Low-density lipoprotein.

## Discussion

In our study, we found that the prevalence of HS in HIV/HCV-coinfected patients is similar to that observed in of HCV-monoinfected patients. These results suggest that HIV coinfection does not influence the development of HS in patients with chronic hepatitis C.

The prevalence of NAFLD in HIV infection, as estimated with CAP, has ranged from 39% to 41% across different independent reports in Western countries^14,15,22^. In the general population, NAFLD frequency is expected to affect 25% individuals living in the USA^5^. Despite this sharp difference between the HIV-infected population and the general population, direct comparisons of large samples of unselected patients with and without HIV infection are lacking. In a recent retrospective study, the frequency of HS, evaluated by CAP, was higher among HCV-monoinfected individuals than HIV/HCV-coinfected patients^23^. The prevalence of CAP ≥ 238 dB/m was 29.5% for HIV/HCV-coinfected patients and 42.9% for HCV-infected patients^23^. However, a comparative analysis of risk factors for HS was not reported^23^. Thus, it is not possible to unequivocally interpret these results as an effect of HIV infection or as the consequence of a different distribution of metabolic risk factors. In addition, the study population reported by Samson *et al*. was mainly African American^23^. Given the distinct effect of ethnicity on the likelihood of NAFLD^24^, it is not possible to extrapolate the results by Sansom *et al*. to populations with other ethnic background, as our Caucasian study patients.

In the present study, contrary to all expectations, the rates of HS, measured by CAP, among patients with HCV infection followed at a single tertiary care center were similar among those with and without HIV coinfection. However, there were a number of differences between both groups that could have influenced the frequency of HS. Notably, BMI and other metabolic factors were unevenly represented in both groups. The HCV-monoinfected group showed a greater proportion of overweight or obese patients, whereas those with HIV/HCV coinfection presented higher levels of plasma triglycerides and lower levels of plasma HDL-cholesterol. After adjustment for factors associated with HS, including those mentioned before, HIV infection was not a factor related with a higher likelihood of steatosis. Indeed, a stratified analysis by BMI, the strongest predictor of HS in the present study and in previous reports^14,15,17,22^, did not disclose any differences in the rates of steatosis by BMI category between patients with and without HIV infection. Finally, an analysis excluding patients with alcohol intake ≥50 g/day did not show either significant differences between both groups. Because of all the above reasons, the risk of NAFLD seems to be similar for patients with chronic hepatitis C with and without HIV infection.

The present study may have several limitations. First, this was a retrospective study and that design may involve lack of data unplanned to be gathered in clinical practice, as insulin resistance. However, all patients with HCV infection attended at our unit undergo the same protocol, including assessment of steatosis by CAP, at their initial clinical visit. CAP data was not available only among individuals in whom images could not be acquired. Second, alcohol intake was self-referred by patients during the clinical interview, and this could underestimate the true amount of alcohol consumption. Third, HS in the present study could represent a mixture of causes, from true NAFLD associated with metabolic factors to secondary steatosis related with HCV genotype 3 infection. Despite this, the main factors associated with HS in the present study were metabolic factors, those typically associated with NAFLD. The main strength of this study is the comparison of the prevalence of and factors associated with HS in a homogeneous population within the same unit, according to the same protocol and evaluated using a uniform technique throughout the study. A study of this kind has been claimed before by some experts^9,10^.

In conclusion, HS is very frequent in patients with chronic hepatitis C, with and without HIV coinfection. Among them, HS shows features of NAFLD, as it is mainly associated with components of metabolic syndrome, and is also related with HCV genotype 3 infection. Our findings indicate that HIV coinfection is not associated with a higher risk of HS in individuals with chronic hepatitis C background. Because of these, the management of HS in HIV/HCV-coinfected patients and HCV-monoinfected patients should be similar and aimed at controlling metabolic risk factors.

## Methods

### Patients and study design

This was a retrospective cross-sectional study. All Spanish Caucasian patients who were attended at the Unit of Infectious Diseases of the Hospital Universitario Virgen de Valme, Seville (Spain), from November 2010 to March 2019, were selected if they had: (1) Chronic HCV infection, with persistent detection of plasma HCV RNA, with or without HIV coinfection; (2) A valid available hepatic elastography examination with evaluation of HS by CAP. Patients pretreated against HCV infection who did not achieve sustained virological response (SVR) were also included in the study.

### Data collection

Data from all patients were recorded following a pre-specified protocol before starting HCV therapy. At that date, electronic clinical records including demographics, self-referred alcohol intake by patients, anthropometry, blood test and hepatic transient elastometry with CAP were gathered. CAP and liver stiffness (LS) were measured by FibroScan (Echosens FibroScan 502, Paris). A cut-off of ≥248 dB/m and of ≥280 dB/m were selected to define the presence of mild HS (steatosis involving ≥10% of hepatocytes) and severe steatosis (≥66% steatotic hepatocytes), respectively^25^. All CAP and LS measurements were performed in fasting conditions by two trained operators. We had previously proven a high concordance between two trained operators in FibroScan measurements, to determine HS by CAP^26^ or to evaluate LS^27^. All the measurements were the result of the evaluation of ten valid shots. For the present study, an hepatic transient elastometry was considered as valid if the interquartile range for liver stiffness was <30% of the median value and the success rate was ≥60%^14^.

Individuals with a body mass index (BMI) between 18 and 25 kg/m^2^, between 25 and 30, between 30 and 35, between 35 and 40 and >40 were considered as individuals with normal weight, pre-obesity, obesity class 1, class 2 and class 3 respectively in accordance with the WHO classification (http://www.euro.who.int/en/health-topics/disease-prevention/nutrition/a-healthy-lifestyle/body-mass-index-bmi). We consider overweight a BMI up to 25 kg/m^2^. High alcohol intake was defined as ≥50 g/day^28^.

### Statistical analysis

For descriptive analysis, continuous variables were expressed as median (Q1–Q3) and categorical variables as frequencies (percentage). The χ^2^ test or Fisher’s exact test was used to compare the distribution of categorical variables between groups and Student’s t-test or the Mann-Whitney U test was used for continuous variables. Binary logistic regression models were elaborated to assess the factors independently associated with the presence of HS. In those analyses, variables related to this condition with a univariate p value < 0.2, as well as age, sex and HIV infection, were included to obtain odds ratio (OR) values. Differences were considered significant for p values < 0.05.

A case-control study was carried out as secondary analysis. HIV/HCV-coinfected patients were considered cases. HCV-monoinfected patients were matched with cases by age, sex and BMI. Cases and controls were matched by BMI because it was the only independent predictor of HS in a previous study using CAP^14^. All analysis were carried out using the SPSS software 25.0 (IBM Corporation, Somers, New York, New York, USA).

### Ethics

This study was designed and performed according to the Helsinki declaration and was approved by the ethics committee of the Hospital Universitario Virgen de Valme (Seville, Spain). Informed consent was obtained from all individuals.

## Supplementary information

Supplementary information.

## Data Availability

All data generated or analyzed during this study are included in this published article (and its Supplementary Information Files).

## References

[1] Yki-Järvinen H (2014). Non-alcoholic fatty liver disease as a cause and a consequence of metabolic syndrome. Lancet Diab. Endocrinol..

[2] Takahashi H (2015). Biphasic effect of alcohol intake on the development of fatty liver disease. J. Gastroenterol..

[3] Goossens N, Negro F (2015). Insulin Resistance, Non-alcoholic Fatty Liver Disease and Hepatitis C Virus Infection. Rev. Recent Clin. Trials.

[4] Leandro G (2006). Relationship Between Steatosis, Inflammation, and Fibrosis in Chronic Hepatitis C: A Meta-Analysis of Individual Patient Data. Gastroenterology.

[5] Diehl AM, Day C (2017). Cause, Pathogenesis, and Treatment of Nonalcoholic Steatohepatitis. N. Engl. J. Med..

[6] Younossi ZM (2016). Global epidemiology of nonalcoholic fatty liver disease-Meta-analytic assessment of prevalence, incidence, and outcomes. Hepatology.

[7] Anstee QM, Targher G, Day CP (2013). Progression of NAFLD to diabetes mellitus, cardiovascular disease or cirrhosis. Nat. Rev. Gastroenterol. Hepatol..

[8] Chalasani N (2018). The diagnosis and management of nonalcoholic fatty liver disease: Practice guidance from the American Association for the Study of Liver Diseases. Hepatology.

[9] Maurice JB (2017). Prevalence and risk factors of nonalcoholic fatty liver disease in HIV-monoinfection. AIDS.

[10] van Welzen BJ, Mudrikova T, El Idrissi A, Hoepelman AIM, Arends JE (2019). A Review of Non-Alcoholic Fatty Liver Disease in HIV-Infected Patients: The Next Big Thing?. Infect. Dis. Ther..

[11] Demir M, Lang S, Steffen H-M (2015). Nonalcoholic fatty liver disease - current status and future directions. J. Dig. Dis..

[12] Rafiq N (2009). Long-Term Follow-Up of Patients With Nonalcoholic Fatty Liver. Clin. Gastroenterol. Hepatol..

[13] Vernon G, Baranova A, Younossi ZM (2011). Systematic review: the epidemiology and natural history of non-alcoholic fatty liver disease and non-alcoholic steatohepatitis in adults. Aliment. Pharmacol. Ther..

[14] Macías J (2014). Prevalence and factors associated with liver steatosis as measured by transient elastography with controlled attenuation parameter in HIV-infected patients. AIDS.

[15] Mohr R (2018). Return-to-health effect of modern combined antiretroviral therapy potentially predisposes HIV patients to hepatic steatosis. Medicine (Baltimore)..

[16] Sulyok M (2015). Hepatic steatosis in individuals living with HIV measured by controlled attenuation parameter: a cross-sectional study. Eur. J. Gastroenterol. Hepatol..

[17] Vuille-Lessard É (2016). Nonalcoholic fatty liver disease diagnosed by transient elastography with controlled attenuation parameter in unselected HIV monoinfected patients. AIDS.

[18] Price JC (2014). Risk factors for fatty liver in the Multicenter AIDS Cohort Study. Am. J. Gastroenterol..

[19] Aepfelbacher, J. A. *et al*. Increased Prevalence of Hepatic Steatosis in Young Adults with Life-long HIV. *J. Infect. Dis*., 10.1093/infdis/jiz096 (2019). 10.1093/infdis/jiz096 PMC6581896 30852587

[20] Vodkin I, Valasek MA, Bettencourt R, Cachay E, Loomba R (2015). Clinical, biochemical and histological differences between HIV-associated NAFLD and primary NAFLD: a case-control study. Aliment. Pharmacol. Ther..

[21] Machado MV, Oliveira AG, Cortez-Pinto H (2010). Hepatic steatosis in patients coinfected with human immunodeficiency virus/hepatitis C virus: A meta-analysis of the risk factors. Hepatology.

[22] Pembroke T (2017). Hepatic steatosis progresses faster in HIV mono-infected than HIV/HCV co-infected patients and is associated with liver fibrosis. J. Hepatol..

[23] Sansom SE (2019). Steatosis Rates by Liver Biopsy and Transient Elastography With Controlled Attenuation Parameter in Clinical Experience of Hepatitis C Virus (HCV) and Human Immunodeficiency Virus/HCV Coinfection in a Large US Hepatitis Clinic. Open Forum Infect. Dis..

[24] Browning JD (2004). Prevalence of hepatic steatosis in an urban population in the United States: impact of ethnicity. Hepatology.

[25] Karlas T (2017). Individual patient data meta-analysis of controlled attenuation parameter (CAP) technology for assessing steatosis. J. Hepatol..

[26] Recio E (2013). Interobserver concordance in controlled attenuation parameter measurement, a novel tool for the assessment of hepatic steatosis on the basis of transient elastography. Eur. J. Gastroenterol. Hepatol..

[27] Neukam K (2010). Interobserver concordance in the assessment of liver fibrosis in HIV/HCV-coinfected patients using transient elastometry. Eur. J. Gastroenterol. Hepatol..

[28] Corrao G, Aricò S (1998). Independent and combined action of hepatitis C virus infection and alcohol consumption on the risk of symptomatic liver cirrhosis. Hepatology.

