# Supplementary material for: Similar prevalence of hepatic steatosis among patients with chronic hepatitis C with and without HIV coinfection
Source: Sci Rep. 2020 Apr 21;10:6736. doi: 10.1038/s41598-020-62671-y (PMC7174281; doi:10.1038/s41598-020-62671-y)
Supplement: Supplementary file 1 — Supplementary information. [file 41598_2020_62671_MOESM1_ESM.pdf]

Similar prevalence of hepatic steatosis among patients with chronic hepatitis C with and without HIV coinfection

Fernandez-Fuertes M[+], Macías J*[+], Corma-Gómez A, Rincón P, Merchante N, Gómez-Mateos J, Pineda JA[+], Real LM[+]

Unit of Infectious Diseases and Microbiology, Hospital Universitario Virgen de Valme, Sevilla, 41014, Spain

*Corresponding author: juan.macias.sanchez@gmail.com

[+]These authors contributed equally to this work

Supplementary Fig. S1: CAP median and interquartile range in HCV-infected and HIV/HCV coinfected populations (A) and frequency of steatosis and severe steatosis in both groups (B) when patients who reported alcohol intake were excluded.

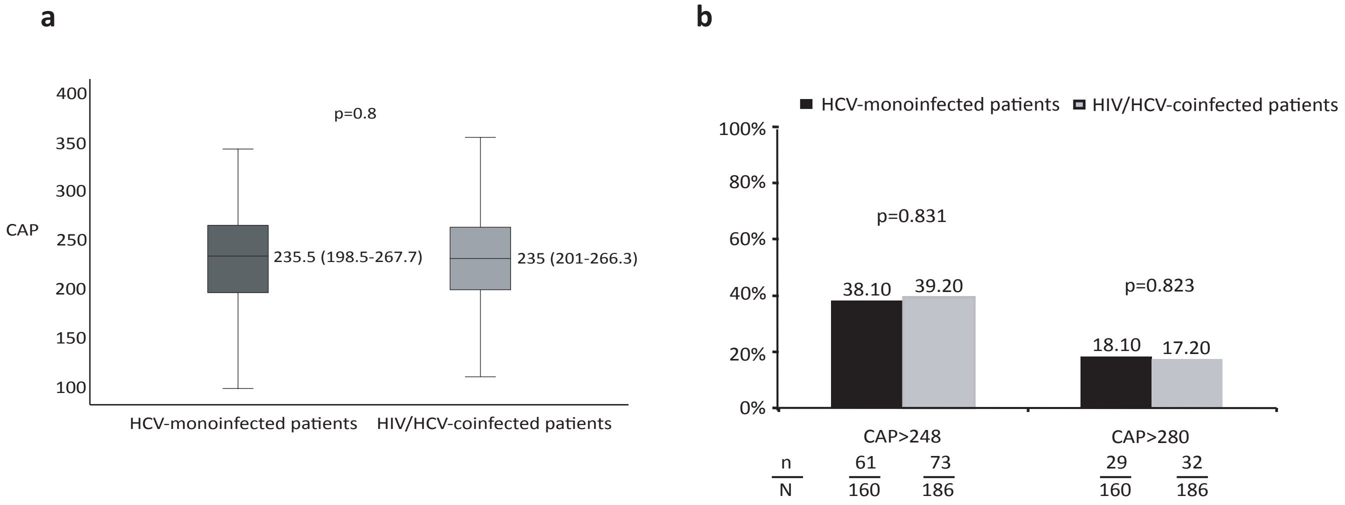

Supplementary Table S1. Characteristics of case-control study populations (N=412)

**Characteristics**	**HCV monoinfection (Controls, n=246)**	**HIV/HCV coinfection (Cases, n=246)**	**p-value****
Male sex, n (%)	206 (83.7)	214 (87)	1.000
Age (years)*	52 (48-56)	53 (49-56)	0.167
IDU [a], n (%)	162 (65.9)	209 (87.1)	0.000
Alcohol intake ≥50 g/d, n (%)	115 (46.7)	60 (24.4)	0.000
BMI [b] (kg/m^2)*	24.03 (21.4-27.1)	24 (21.3-27)	0.361
Fasting plasma glucose [c] ≥100 mg/dL, n (%)	40 (16.3)	75 (30.5)	0.013
Plasma triglycerides [d] ≥150 mg/dL, n (%)	22 (8.9)	82 (33.5)	0.000
Plasma total cholesterol [e] (mg/dL)*	167 (143-196)	156 (137-182)	0.013
Plasma HDL-cholesterol [f] (mg/dL)*	52 (41.8-67.1)	47.5 (37.6-59.0)	0.015
Plasma LDL-cholesterol [g] (mg/dL)*	90 (67.8-116)	76 (61.0-99.0)	0.002
LS (kPa)*	7.1 (4.9-12.8)	9.7 (6.9-16.8)	0.001
Cirrhosis, n (%)	55 (22.4)	80 (32.5)	0.042

* *Median (Q1-Q3)*

** *continuous variables were compared using Wilcoxon signed-rank test and categorical variables using McNemar's test*

Abbreviations: HCV: hepatitis C virus; HIV: human immunodeficiency virus; IDU: injecting drug users; BMI: Body mass index; HDL: High-density lipoprotein; LDL: Low-density lipoprotein; LS: Liver Stiffness.
